# Structural modification of egg white proteins enhances electrostatic complexation with sodium alginate and hydrogel particle formation: mechanistic insights and functional applications

**DOI:** 10.1016/j.crfs.2026.101339

**Published:** 2026-02-03

**Authors:** Fan Zhang, Hangxin Zhu, Fangfang Li, Bongkosh Vardhanabhuti, Azlin Mustapha, Yujie Su, Yanjun Yang, Zipei Zhang

**Affiliations:** a Food Science Program, Division of Food, Nutrition & Exercise Sciences, University of Missouri, Columbia, MO, 65211, USA; b State Key Laboratory of Food Science and Technology, School of Food Science and Technology, Jiangnan University, Wuxi, Jiangsu, 214122, PR China

**Keywords:** *Egg white protein*, *Hydrogel particles*, *Fish oil emulsion*, *Structural modification*

## Abstract

Many bioactive compounds, such as fish oil, are prone to oxidation, which limits their incorporation into functional foods. Egg white proteins (EWP) have notable antioxidant properties, making them promising candidates for stabilizing such compounds, but their compact globular structure limits flexibility and reduces their ability to form stable hydrogel particles with polysaccharides. Based on this, the purpose of this study was to explore the structural modification of EWP via pH-shifting and heat treatment to enhance their electrostatic complexation with sodium alginate (NaAlg) and subsequent hydrogel particle formation. We compared different modification treatments and found that treatment at pH 13 and 50 °C produced the optimal effect, effectively exposing tryptophan and tyrosine residues and increasing the structural flexibility of EWP while maintaining its ionization properties. These conformational changes promoted strong electrostatic interactions with NaAlg, resulting in uniformly distributed hydrogel particles. Confocal microscopy confirmed the encapsulation of fish oil emulsion within the hydrogel particles, and the formed particles exhibited improved oxidative stability and dispersion compared to simple dispersions. These enhancements were attributed to both the physical encapsulation within the hydrogel matrix and the intrinsic antioxidant properties of EWP. This work demonstrates how increasing protein flexibility can facilitate polysaccharide complexation and hydrogel formation, providing mechanistic insights and guiding potential applications for compact globular proteins in functional food systems.

## Highlights

• Egg white proteins (EWP) were modified via pH-shifting and heat treatment.
• Hydrophobicity and structural flexibility of EWP increased after modification.
• Modified EWP and NaAlg formed uniform, stable hydrogel particles.
• Fish oil emulsions could be encapsulated within the MEWP-rich particles.
• Filled hydrogel particles improved the oxidative and dispersion stability of the encapsulated fish oil emulsion compared to a simple emulsion.

## Graphical abstract

**Figure:**
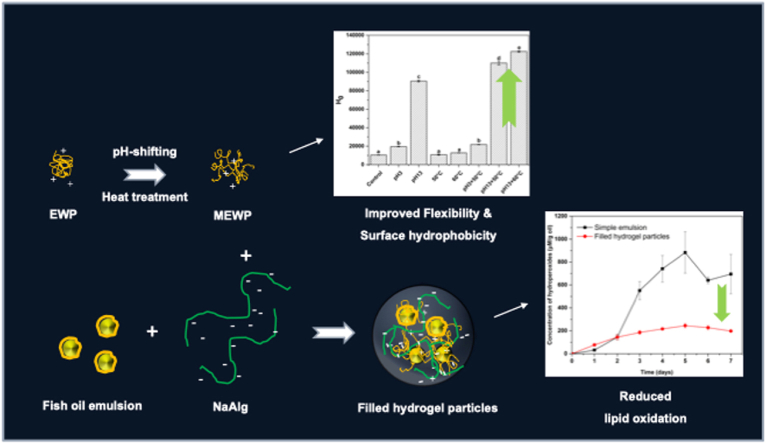

## 1 Introduction

Electrostatic interactions between proteins and polysaccharides are fundamental in shaping the texture, stability, and functional performance of many food systems, while also allowing the creation of innovative food structures with improved properties and applications. Hydrogel particles are a typical example of such electrostatic complexes, where oppositely charged proteins and polysaccharides electrostaticly interact with each other to form a three-dimensional network that traps water, resulting in discrete particles dispersed within a continuous biopolymer phase (McClements and Li, 2010). Hydrogel particles can be used to encapsulate, protect, and release bioactive agents such as nutraceuticals, cosmetics, and pharmaceuticals. Additionally, they can be utilized in food products to modulate the appearance, rheology, and stability of aqueous solutions by scattering light, altering fluid flow, and increasing viscosity. The formation and properties of these hydrogels largely depend on the structure of the proteins, as all proteins in the system contribute to the formation of the dispersed particle phase, while only a portion of the polysaccharides participate in particle formation, with the majority remaining in the continuous phase (Wijaya et al., 2017). Therefore, hydrogel particles can be regarded as a protein-enriched phase, which effectively amplifies the functionality of proteins. In particular, when proteins exhibit antioxidant activity, these particles provide not only physical protection to the encapsulated components but also additional chemical protection.

In the egg processing industry, many foods (such as mayonnaise, baking mixes, sauces, ice cream) mainly use egg yolks, resulting in a large amount of egg white by-products being left over during the production process (Xiao et al., 2021). Egg white protein (EWP) is a substance of exceptionally high nutritional value. EWP, composed mainly of 54% ovalbumin, 12% ovotransferrin, 11% ovomucoid, 3.5% ovomucin, and 3.4% lysozyme, are promising building blocks for the formation of hydrogel particles due to their notable antioxidant activity (Benede and Molina, 2020; Razi et al., 2023). Utilizing EWP for the development of high-value hydrogel particles also aligns with the principles of the circular economy, as it enables the valorization of food industry co-products and minimizes waste streams. Such approaches contribute to more sustainable food production practices and enhance resource efficiency within the broader agri-food system.

While EWP are promising, their native globular structure limits their functionality in forming such complexes, necessitating modification. Effective modification strategies that facilitate EWP-based hydrogel formation for encapsulating easily oxidized compounds such as fish oil could not only enhance oxidative stability but also reduce dependence on synthetic antioxidants, promoting clean-label production and more efficient utilization of natural resources. Among various approaches, physical modification methods are particularly attractive, as they can induce conformational changes in EWP that alter hydrophilicity and structural flexibility, thereby facilitating hydrogel particle formation. Heating and pH adjustment are among the most common and effective approaches to enhance the emulsifying properties of egg white protein. For example, Chang et al. (2016) demonstrated that acid treatment combined with heating improved the emulsifying capacity of egg white protein, as acid and heat treatment can induce tertiary structural transitions and thereby extend the storage stability of emulsions. Similarly, under alkaline conditions, the content of free sulfhydryl groups in egg white protein can be increased, leading to protein unfolding and the exposure of phenylalanine/tyrosine and tryptophan residues to the polar environment, which in turn improves emulsion stability (Yu et al., 2021).

Fish oil is a representative polyunsaturated lipid primarily composed of omega-3 fatty acids, such as eicosapentaenoic acid (EPA) and docosahexaenoic acid (DHA) (Erbay et al., 2024). These long-chain polyunsaturated fatty acids are well known for their diverse health-promoting properties, including anti-inflammatory activity, cardiovascular protection, and potential cognitive benefits (Liao et al., 2022). However, despite these recognized advantages, the utilization of fish oil in food applications remains limited due to its poor water dispersibility and high susceptibility to oxidative degradation. In this study, we used different methods, pH-shifting and/or mild heat treatments, to modify the EWP and alter its conformational structure and hydrophilicity. Hydrogel particles were then prepared using modified EWP (MEWP) and sodium alginate (NaAlg) through electrostatic complexation. The resulting filled hydrogel particles, formed after encapsulating the fish oil emulsion, were subsequently characterized, and their protective effect against the lipid oxidation of fish oil was evaluated. By improving the oxidative stability of sensitive nutrients such as omega-3 fatty acids, this approach not only enhances the nutritional quality and health profile of food products but also supports nutrition security by preserving essential bioactive compounds during processing and storage. Furthermore, by integrating molecular design with sustainable utilization of food by-products, this study contributes to the development of innovative delivery systems that promote circular resource use, reduce food waste, and strengthen the link between food technology innovation and global sustainability goals. The results of this study provide valuable insights into the rational design and fabrication of delivery systems for active compounds that are susceptible to oxidation. Additionally, this study was conducted in order to expand fundamental knowledge about the relationship between molecular properties of proteins and the structural properties of electrostatic complexes formed by these proteins in combination with polysaccharides, a relationship that has not been thoroughly explored.

## 2 Materials and methods

### 2.1 Materials

Fresh brown eggs were purchased from Walmart in Columbia (MO, USA). 8-Anilino-1-naphthalenesulfonic acid (ANS) was purchased from Thermo scientific (Waltham, MA, USA). Fish oil from menhaden, NaAlg, fluorescein isothiocyanate isomer I (FITC), Nile Red, cumene hydroperoxide, ammonium thiocyanate, and ferrous chloride were purchased from Sigma-Aldrich (St. Louis, MO, USA). All other chemicals used in this study were of analytical grade.

### 2.2 Preparation of EWP stock solution

The EWP stock solution was prepared using the method described by (Chang et al., 2016) with some modifications. Egg white was manually separated from fresh eggs and adjusted to pH 5 using 1 M HCl. The egg white was stirred at room temperature for 1 h and then centrifuged at 6000 *g* for 15 min at 4 °C. Subsequently, the supernatant was mixed with two volumes of 10 mM phosphate buffer (pH 7) and adjusted to pH 7 with 1 M NaOH. The resulting solution was designated as EWP stock solution for future use. The protein content of EWP stock solution was determined using the Lowry procedure. Briefly, samples were reacted with alkaline copper reagent followed by Folin–Ciocalteu phenol reagent, and the absorbance was measured at 650 nm. Bovine serum albumin (BSA) was used to construct the standard calibration curve.

### 2.3 Preparation of modified EWP (MEWP)

#### 2.3.1 pH-shifting

**Fig. 1:**
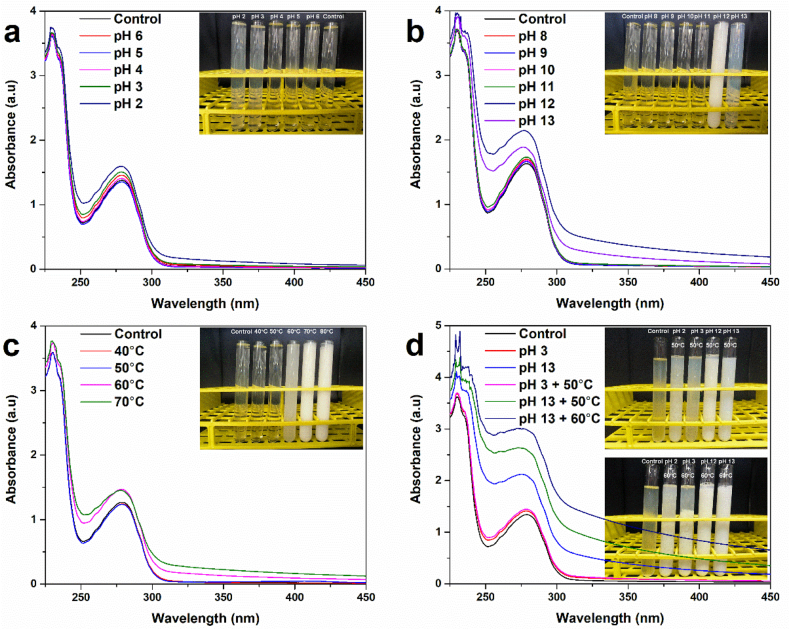
UV-vis spectroscopy and macroscopic images of MEWP prepared by (a-b) pH shifting; (c) Heat treatment; (d) Combined pH shifting and heat treatment.

The pH of EWP stock solution was adjusted to a range of 2 to 13 (all integer pH) using 1 M HCl or 1 M NaOH and held at room temperature for 1 h to induce conformational changes. Afterward, the pH of each sample was adjusted back to pH 7 by adding 1 M HCl or 1 M NaOH. Protein unfolding was analyzed based on UV absorbance spectra. Based on the results (Fig. 1 ab), UV absorbance spectra recorded within the pH range of 2–13 showed that samples at pH 2, 3, 12 and 13 exhibited consistently higher absorbance across the entire wavelength range compared with intermediate pH conditions, indicating a greater extent of protein unfolding. Therefore, pH 2, 3, 12, and 13 were selected as representative groups for subsequent experiments.

#### 2.3.2 Heat treatment

A 30 mL aliquot of EWP stock solution was heated at 40, 50, 60, 70 or 80 °C for 10 min using an Isotemp 2231 water bath (Fisher Scientific, Waltham, MA, USA). The heated EWP solutions were then cooled to room temperature and shaken well before further analysis.

#### 2.3.3 The combined pH-shifting and heat treatment

EWP stock solution was adjusted to pH 2, 3, 12 or 13, held at room temperature for 1 h, and then adjusted back to pH 7. After pH-shifting, 30 mL of EWP solution was heated at 50 or 60 °C for 10 min and then cooled to room temperature.

### 2.4 UV-vis spectroscopy

The measurements of UV-vis spectroscopy were conducted using the method described by (Zhang et al., 2020). The absorption values of different EWP solutions (1.5 mg/mL) were recorded from 225 to 450 nm with a 1 cm cuvette using a GENESYS 150 UV-Visble Spectrophotometer (Thermo scientific, Waltham, MA, USA). The EWP stock solution (1.5 mg/mL) was used as the control.

### 2.5 Surface hydrophobicity (H_0_)

Surface hydrophobicity was determined according to the method described by (Y. Yu et al., 2021; Chen et al., 2011), employing ANS as a fluorescence probe. Different EWP samples were diluted with 10 mM phosphate buffer (pH 7) to concentrations of 0.01%, 0.02%, 0.05%, 0.1%, 0.2% and 0.5%. Next, 20 μL of 8 mM ANS solution was added to 4 mL of each sample, vortexed for 15 s, and kept in the dark for 10 min. The fluorescence intensity of the mixture was measured using a SYNERGY H1 microplate reader (BioTek, Winooski, VT, USA) at an excitation wavelength of 390 nm and an emission wavelength of 470 nm. A curve of fluorescence intensity versus protein concentration was constructed, and the initial slope of the curve represents the surface hydrophobicity (H_0_).

### 2.6 Nile Red fluorescence

The protein aggregation state was assessed using Nile Red fluorescence, following the method described by (Sante-Lhoutellier et al., 2008). Briefly, 30 μL of Nile Red solution (0.32 mg/mL in ethanol) was added to 3 mL of 1 mg/mL EWP solution. After shaking, 200 μL of mixture was transferred to a 96-well fluorescence plate and kept in the dark for 10 min. The Nile Red fluorescence of the mixture was measured at an excitation wavelength of 560 nm and an emission wavelength of 620 nm using a microplate reader.

### 2.7 Zeta potential

Zeta potential of EWP was measured at two defined pH conditions (pH 7.0 and pH 4.5). Briefly, the EWP dispersions were first adjusted to pH 7.0 or pH 4.5 using 1 M NaOH or 1 M HCl, respectively, and allowed to equilibrate for 15 min. Prior to measurement, each pH-adjusted sample was diluted with 10 mM phosphate buffer that had been pre-adjusted to the same pH (pH 7.0 or pH 4.5) to maintain the target pH and to minimize multiple scattering. The zeta potential was then determined at 25 °C using a Zetasizer Nano ZS (Malvern, Worcestershire, UK) equipped with a DTS1070 folded capillary cell. Measurements were performed in triplicate under automatic voltage mode, and the Smoluchowski model was used to calculate the Zeta potential from electrophoretic mobility.

### 2.8 Preparation of oil-in-water (O/W) emulsion, unfilled and filled hydrogel particles

The emulsifier solution was prepared by diluting EWP stock solution to 1% (w/w) with 10 mM phosphate buffer (pH 7). Fish oil was mixed with the emulsifier solution at a mass ratio of 1:9, with continuous stirring at 600 rpm for 10 min. The mixture was then homogenized using a Homogenizer 850 (Fisher brand, Waltham, MA, USA) at 10000 rpm for 2 min. After homogenization, the droplet size of the emulsion was further reduced by passing it three times through an LM20 Microfluidizer (Microfluidics, Westwood, MA, USA) at 12,000 psi. The resulting product was a 10% (w/w) O/W emulsion.

The unfilled and filled hydrogel particles were prepared based on our previous methods with some modifications (Zhang et al., 2014). For the unfilled hydrogel particles, 3% (w/w) MEWP (pH 7) was mixed with an equal volume of 3% (w/w) NaAlg (pH 7, in 10 mM phosphate buffer) and stirred for 10 min at 300 rpm. The pH of the mixture was adjusted to pH 4.5 using 0.1 M citric acid. The resulting product was designated as unfilled hydrogel particles.

For the fabrication of filled hydrogel particles, 3% (w/w) MEWP, 10% (w/w) O/W emulsion, and 10 mM phosphate buffer were mixed at a weight ratio of 5:1:4, yielding a system containing 1.5% (w/w) MEWP and 1% (w/w) fat. Then this droplet-biopolymer mixture was mixed (300 rpm) with an equal volume of 1.5% (w/w) NaAlg to form an oil-in-water-in-water (O/W_1_/W_2_) emulsion to get the final system has 0.75% MEWP, 0.5% fat, and 0.75% NaAlg. The pH of the mixture was adjusted to pH 4.5 using 0.1 M citric acid to promote complex formation. The resulting product was designated as filled hydrogel particles.

### 2.9 Microstructure analysis

The microstructure of different systems was observed using optical microscopy with 40 × and 100 × objective lenses. The distribution of protein and oil in the systems was analyzed using confocal scanning laser microscopy (Leica TCS SP8, Danaher, Washington, D.C., USA). To stain the protein phase and oil phase, 20 μL of FITC solution (1 mg/mL in dimethyl sulfoxide) and 20 μL of Nile Red solution (1 mg/mL in ethanol) were added to 1 mL of the samples, respectively. After mixing, the microstructure of samples was observed with a 40 × objective lens and 10 × eyepiece. The excitation and emission spectra for FITC were 488 nm and 515 nm, respectively, while for Nile Red they were 543 nm and 605 nm, respectively.

### 2.10 Particle size measurements

The particle sizes of samples were determined using a Mastersizer 3000 (Malvern, Worcestershire, UK) based on static light scattering. Different samples were added to a measurement chamber filled with the corresponding sample solvent to reach an intensity of 10 %. A refractive index of 1.33 was used for the dispersant phase and 1.472 for the material phase. Particle size measurements were reported as volume weighted (D_43_) or surface weighted (D_32_) mean diameters.

### 2.11 Determination of lipid oxidation

The chemical stability of fish oil in the filled hydrogel particles (encapsulated samples) and in the emulsion (non-encapsulated samples) was evaluated by determining the formation of hydroperoxides over a 7-day storage period at 37 °C, following our previous methods (Zhang et al., 2014). The hydroperoxides in 1 mL of samples were extracted with 4 mL of chloroform/methanol (2:1, v/v) solution, followed by centrifugation for 5 min at 1300 g. Then, 200 μL of extract from the lower layer was pipetted and mixed with 2.8 mL of methanol/1-butanol (2:1, v/v). Subsequently, 15 μL of 3.94 M ammonium thiocyanate and 15 μL of ferrous chloride solution were added to the mixture. After vortexing, the mixture was allowed to react at room temperature in the dark for 20 min. The absorbance of the mixture was measured at 510 nm using a spectrophotometer and the concentration of hydroperoxides was determined based on a standard curve of cumene hydroperoxide (0–100 μM).

### 2.12 Statistical analysis

All experiments were performed in triplicate unless otherwise stated, and the obtained data were expressed as the means ± standard deviations and analyzed by SPSS 20.0 using one-way analysis of variance (ANOVA), followed by Tukey's test. A level of *p* < 0.05 was set as significance threshold between groups.

## 3 Results and discussion

### 3.1 The modification of EWP by pH-shifting or/and heat treatment

Globular proteins have compact, spherical structures stabilized by electrostatic and some weak forces (e.g., hydrogen bonds, van der Waals forces, and hydrophobic interactions). These forces maintain the three-dimensional structure of protein. In the native state, proteins preferentially orient hydrophilic groups towards the aqueous environment, while folding hydrophobic groups within their interior, thereby maximizing thermodynamic stability (Onuchic and Wolynes, 2004). However, globular proteins are sensitive to environmental changes, especially pH, which can alter their compactly folded structure (Cortes-Morales et al., 2021). As shown in Fig. 1a and b, the UV-vis spectroscopy of EWP underwent significant changes after pH-shifting treatments, with the most notable alterations observed in the samples treated at pH 12 and 13. These results are consistent with the increased turbidity observed in the EWP solutions, as shown in the macroscopic images (inset of Fig. 1b). The UV absorption intensity of EWP at 280 nm increased from 1.62 to 1.84 (pH 13) and 2.11 (pH 12) after treatment at pH 13 and 12, respectively. The increased UV absorbance of EWP at 280 nm is associated with greater exposure of the aromatic amino acids tryptophan and tyrosines. This indicates that our pH-shifting treatments can partially unfold the compact globular EWP protein, exposing previously buried hydrophobic regions and potentially enhancing the protein's structural flexibility. In our study, the UV-vis spectra of the different EWP were obtained after readjusting to pH 7, indicating that the conformational changes induced by pH-shifting are irreversible.

The absorption intensity of EWP was increased when the treating temperature reached 60 °C (Fig. 1c), indicating that heat treatment can also induce conformational changes in EWP. Further raising the temperature to 70 °C resulted in the formation of flocculent precipitates, and the EWP solution transitioned to a semi-solid state at 80 °C (inset of Fig. 1c). To assess the combined modification effects of pH-shifting and heat treatment on EWP, samples treated at pH 2, 3, 12, and 13 were further subjected to heat treatment at 50 °C and 60 °C. UV-Vis spectroscopy results demonstrated that the combined treatment caused more pronounced changes in absorption intensity compared to either pH-shifting or heat treatment alone (Fig. 1d). This suggests that EWP becomes more sensitive to heat treatment following pH-shifting, and that the combined treatments (i.e., heating after pH-shifting) can further promote conformational changes in EWP, leading to the combined treatments of pH 3 + 50 °C, pH 13 + 50 °C, and pH 13 + 60 °C remained physically stable in solution, as shown in macroscopic images, indicating their potential for hydrogel particle formation. Notably, although pH 12 resulted in a higher absorbance at 280 nm than the pH 13 treatment (Fig. 1b), subsequent heating drove EWP (pH 12 + 50 °C and pH 12 + 60 °C) into a more aggregation-prone state (Fig. 1d inset pictures). This discrepancy may arise because pH 13 induces a deeper yet more controlled unfolding that becomes partially irreversible upon neutralization, yielding a more flexible polypeptide conformation without collapsing into dense aggregates. In contrast, pH 12 may generate more heterogeneous or unstable unfolding intermediates, which can promote intermolecular association and aggregation during subsequent heating (Yu et al., 2021).

### 3.2 Physicochemical properties of EWP modified by pH-shifting or/and heat treatment

**Fig. 2:**
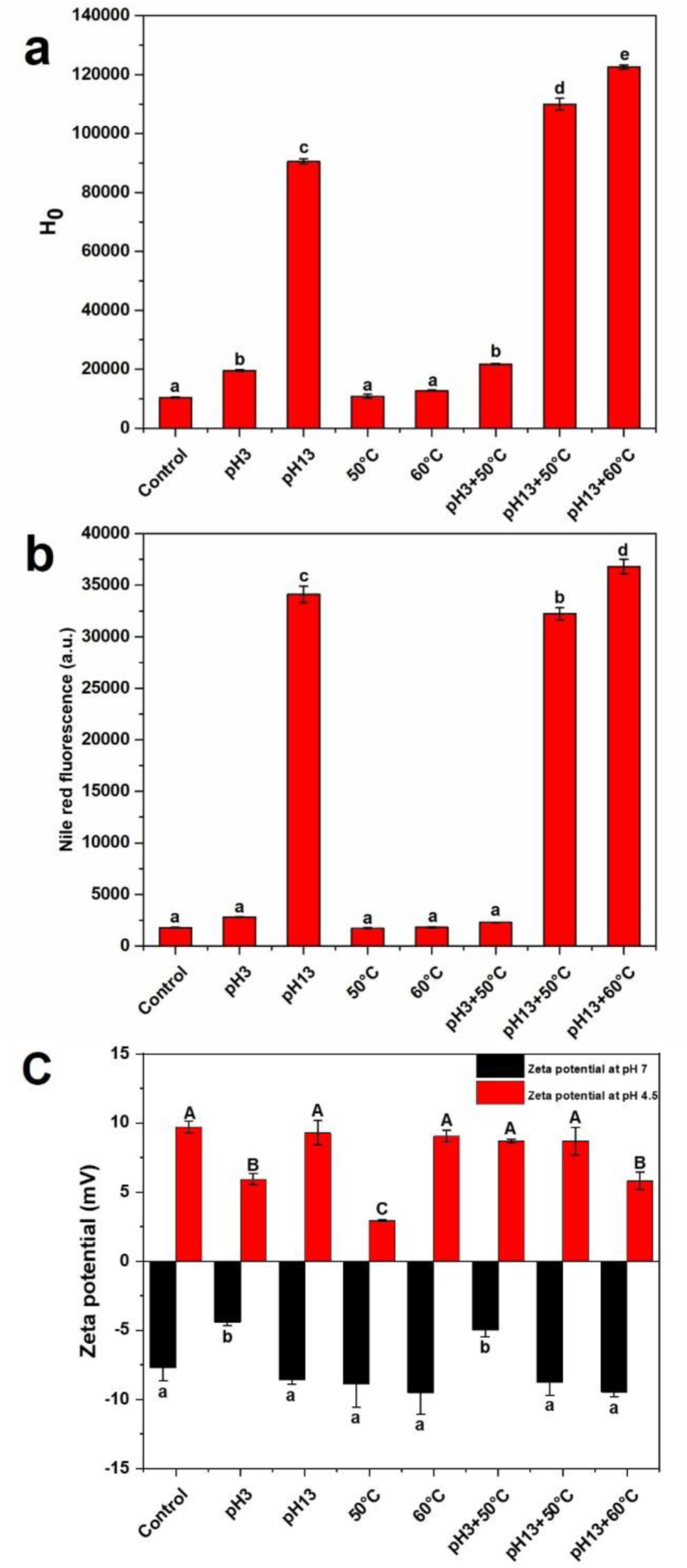
Physicochemical properties of MEWP. (a) Surface hydrophobicity; (b) Nile Red fluorescence; (c) Zeta potential at pH 7 (black) pH 4.5 (red) (Tukey, *p* < 0.05). Capital letters indicate the within-group differences at pH 4.5, while lowercase letters indicate the within-group differences at pH 7 (Note: all error bars are incorporated in Fig. 2, but they are not visually apparent due to their relatively small magnitude).

Surface hydrophobicity (H_0_) represents the presence and distribution of hydrophobic regions on the protein surface, which are typically found within hydrophobic binding pockets in the protein's core when it is in its native state (Nishinari et al., 2014). As shown in Fig. 2a, the surface hydrophobicity of EWP increased significantly after pH-shifting treatments (pH 3 and 13), while the effect of single heat treatment (50 °C or 60 °C) was not significant. The combined treatments also further increased the surface hydrophobicity of EWP, which is consistent with the changes observed in the UV-vis spectroscopy. This increased surface hydrophobicity could be attributed to significant conformational changes in EWP, leading to the exposure of hydrophobic side chain groups that were originally enclosed within its dense globular structure following our treatments.

Protein aggregation was evaluated using Nile Red, which fluoresces more strongly when bound to hydrophobic regions on the protein surface. Its fluorescence increases in aggregates compared to monomers, making it useful for detecting protein aggregation (Demeule et al., 2007). Our results indicate that the changes in the aggregation state of EWP were similar to those observed in its surface hydrophobicity (Fig. 2b), this correlation suggests that the increase in surface hydrophobicity is a major driver of protein aggregation under these treatment conditions.

The surface charge of proteins primarily arises from ionization of surface groups, specifically the acidic and basic side chains of the component amino acids, suggesting that it is influenced by changes in the protein's three-dimensional structure (Bowen et al., 1998). EWP has a reported pI of approximately 4.5–4.8. As shown in Fig. 2c, the zeta potential of native EWP was positive at pH 4.5, indicating that the protein carried a net positive charge below its isoelectric point, which provided the electrostatic driving force for complexation with anionic alginate. The combined pH-shifting and heating treatment slightly decreased the zeta potential of EWP, indicating a reduction in surface charge density following modification, which can be attributed to structural rearrangements and likely promoted the exposure of previously buried carboxyl groups and altered the balance between protonated amino groups and deprotonated carboxyl groups on the protein surface (Yu et al., 2021). In addition, limited aggregation of modified EWP (MEWP) particles might have partially shielded surface charges. Nevertheless, MEWP remained positively charged at pH 4.5, ensuring sufficient electrostatic attraction with negatively charged alginate chains and enabling the formation of stable mEWP–alginate complexes.

### 3.3 Structural characterization of unfilled and filled hydrogel particles

#### 3.3.1 Microstructure

**Fig. 3:**
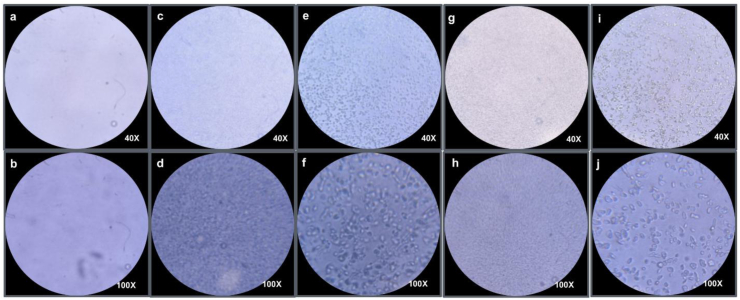
Microscope images of various systems at low and high magnification: Unfilled hydrogel particles prepared using (a-b) native EWP, few and loosely aggregated particles were observed (c-d) pH 13-treated EWP, more particles appeared, but their distribution remained irregular were observed in pH 13-treated EWP (e-f) pH 13 + 50 °C-treated EWP, a larger number of uniformly distributed particle were formed in pH 13 + 50 °C-treated EWP (g-h) 10% O/W emulsion, well-dispersed oil droplets were formed in 10% O/W emulsion (i-j) Filled hydrogel particles prepared using pH 13 + 50 °C-treated EWP, compact and spherical structures.

To identify suitable components for forming hydrogel particles, native EWP and two modified EWP (i.e., pH 13-treated EWP and pH 13 + 50 °C-treated EWP) were selected to investigate their capacity for forming electrostatic complexes with NaAlg at pH 4.5. The temperature of 50 °C was chosen as the optimal condition because it provides comparable protein surface exposure to that achieved at 60 °C while requiring lower energy input. The microstructure of different samples was characterized using optical microscopy and confocal scanning laser microscopy. As shown in Fig. 3a and b, no visible particles were formed in the native EWP-NaAlg mixture at pH 4.5, indicating a one-phase system. It could be speculated that EWP and NaAlg can exist as soluble complexes, resulting in a one-phase system (Matalanis et al., 2011). Previous studies have found that spherical hydrogel particles with a uniform size distribution are most effectively formed by proteins that adopt a flexible and relatively unfolded configuration, such as casein and gelatin (Wu et al., 2014; Zhang et al., 2014, 2015; Zou et al., 2016). However, EWP adopts a less flexible structure due to its polypeptide chains folding into compact globular conformations, which may hinder its ability to extensively complex with polysaccharides via electrostatic interaction and subsequently form hydrogel particles.

With the unfolding of protein structure following pH-shifting or/and heat treatment, hydrogel particles with varying morphologies gradually formed at pH 4.5 (Fig. 3c–f), showing irregular and partially aggregated particles for pH 13-treated EWP (Fig. 3c and d) and more regularly shaped, uniformly sized particles for pH 13 + 50 °C-treated EWP (Fig. 3e and f). The structural properties of unfilled hydrogel particles (Fig. 3e and f) are similar to those of filled ones (Fig. 3i and j), indicating that the morphology and size of the hydrogels did not change when oil droplets were incorporated. Compared to pH-shifting alone, the additional mild heat treatment likely further enhanced molecular mobility and structural rearrangement, allowing the partially unfolded proteins to adopt a more flexible yet still coherent conformation. This balance between unfolding and structural integrity may be critical for effective electrostatic complexation with alginate to form more regularly shaped, uniformly sized particles. This structural state is consistent with a molten globule–like conformation, which is characterized by the retention of substantial secondary structure, loss of native tertiary packing, increased surface hydrophobicity, and enhanced chain flexibility (Van der Plancken et al., 2006; Yu et al., 2021). In our system, the increased exposure of aromatic residues (UV–Vis), elevated surface hydrophobicity (H_0_), and the absence of macroscopic precipitation collectively support the formation of such a partially unfolded, yet structurally intact intermediate state. Proteins in the molten globule state are known to exhibit increased affinity for hydrophobic probes (e.g., ANS), preserved secondary structures as observed by far-UV circular dichroism, and diminished tertiary order as reflected by near-UV CD (Poklar et al., 1997; Semisotnov et al., 1991; Vassilenko and Uversky, 2002). Although these techniques were not employed here, our observations are in strong agreement with these established hallmarks. Importantly, this balance between structural integrity and conformational flexibility appears critical for effective electrostatic complexation with alginate, thereby enabling the formation of more regularly shaped, uniformly sized hydrogel particles.

**Fig. 4:**
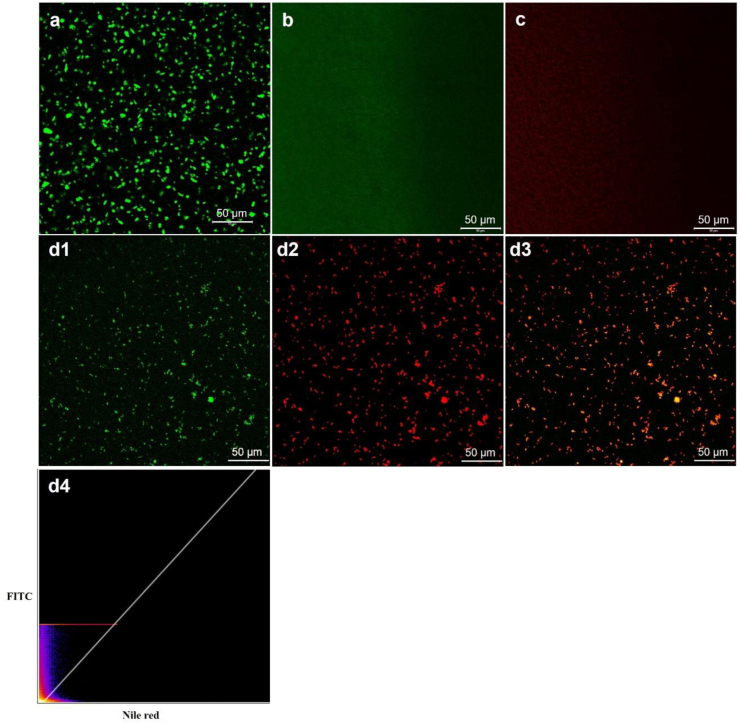
Confocal micrographs of different systems: (a) unfilled hydrogel particles formed by MEWP-NaAlg at pH 4.5, uniformly shaped particles were formed; (b) soluble complex of native EWP–NaAlg at pH 4.5, no particles were observed in native EWP-NaAlg mixture; (c) 10% fish oil emulsion; (d1–d3) filled hydrogel particles containing 10% fish oil emulsion, where (d1) proteins stained with FITC (green), (d2) fish oil stained with Nile Red (red), and (d3) merged image showing colocalization of FITC and Nile Red signals, indicated by orange regions. (d4) two-dimensional intensity histogram of FITC and Nile Red fluorescence signals (Pearson's R value: 0.59).

The confocal images showed that a large number of micrometer-sized hydrogel particles were formed by MEWP and NaAlg at pH 4.5 (Fig. 4a), indicating that majority of MEWP (stained green) participated in hydrogel particles formation. In contrast, no visible micrometer-sized particles were observed when native EWP was used for hydrogel particle fabrication (Fig. 4b), which is consistent with the data obtained from 0 0optical microscopy. For the 10% O/W fish oil emulsion, small lipid droplets (stained red) were distributed throughout the soultion (Fig. 4c). For the filled hydrogel particles, most lipid droplets were embedded inside the MEWP-rich hydrogel particles, forming an O/W_1_/W_2_ structure (Fig. 4d1-3). The filled hydrogel particles appeared yellow-orange in the image when the fluorescence channels of FITC and Nile Red were merged (Fig. 4d3). The merged fluorescence image showed a predominant yellow–orange signal, and quantitative colocalization analysis yielded a Pearson's correlation coefficient of 0.59, suggesting a moderate spatial association between MEWP and lipid droplets, consistent with their incorporation into the MEWP-rich hydrogel particles.

#### 3.3.2 Particle size of unfilled and filled hydrogel particles

**Fig. 5:**
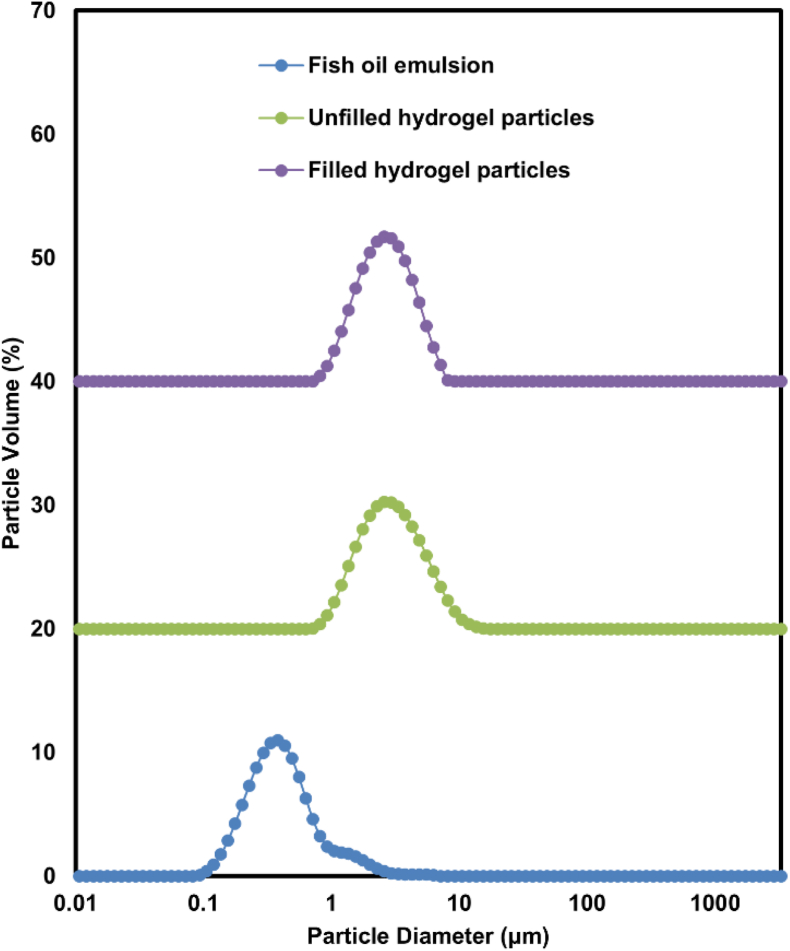
Particle size distributions of different systems including 10% fish oil emulsion, unfilled and filled hydrogel particles prepared using pH 13 + 50 °C-treated EWP.

Knowledge of the particle size of hydrogel particles is essential for ensuring overall functionality in various applications. As shown in Fig. 5, the initial fish oil-in-water emulsion contained relatively small lipid droplets with a single size distribution peak at a particle diameter of about 0.35 μm (D_32_). The particle size distribution of the filled hydrogel particles showed a single peak around 2.9 μm (D_43_), which was even smaller than that of the unfilled hydrogel particles in the absence of lipid droplets (D_43_ = 3.4 μm). As shown in Table .1 the filled hydrogel particles showed the smallest Span value (1.32), indicating the size uniformity of the filled hydrogel particles is superior to that of the unfilled hydrogel particles and the original emulsion.

**Table 1:** Particle size of different systems.

Systems	D_43_ (μm)	D_32_ (μm)	D10 (μm)	D50 (μm)	D90 (μm)	Span
10% O/W fish oil emulsion	0.51 ± 0.02 c	0.35 ± 0.00 c	0.19 ± 0.00c	0.39 ± 0.00c	0.923 ± 0.03c	1.87 ± 0.07
Unfilled hydrogel particles	3.38 ± 0.01 a	2.53 ± 0.01 a	1.44 ± 0.01a	2.87 ± 0.01a	6.03 ± 0.01a	1.60 ± 0.00
Filled hydrogel particles	2.89 ± 0.01 b	2.33 ± 0.01 b	1.39 ± 0.01b	2.61 ± 0.00b	4.85 ± 0.01b	1.32 ± 0.00

Based on these data, the larger size of unfilled particles can be rationalized by the formation of a more loosely crosslinked and highly swollen MEWP hydrogel network in the absence of oil. Previous studies have shown that hydrogels with looser network structures generally exhibit higher water absorption capacity and swelling ratios, which can lead to increased particle dimensions (Feng and Wang, 2023). In contrast, oil incorporation promotes the adsorption of proteins at the oil–water interface, which reduces bulk network formation and results in a denser, less swellable structure in the filled particles. Previous studies have shown that oil tends to concentrate within the protein-rich phase rather than the polysaccharide-rich phase during the formation of filled hydrogel particles. This behavior arises from differences in depletion interactions between the two biopolymer phases. Specifically, the unfavorable depletion interaction—caused by the osmotic pressure gradient generated when biopolymers are excluded from the droplet surfaces—is stronger in the polysaccharide-rich phase than in the protein-rich phase. As a result, proteins preferentially form a shell around the oil droplets, which subsequently interacts with polysaccharides to generate the filled hydrogel particles (Matalanis et al., 2010). In this process, the oil droplets likely act as pre-formed templates, limiting the amount of water that can be incorporated into the particle core and leading to a denser, less swollen network in the resulting filled particles. The size of the hydrogel particles can be tailored by adjusting the shear force. Smaller sizes may hinder the incorporation of submicron O/W emulsions, while larger sizes may affect the stability of the hydrogel particles.

**Fig. 6:**
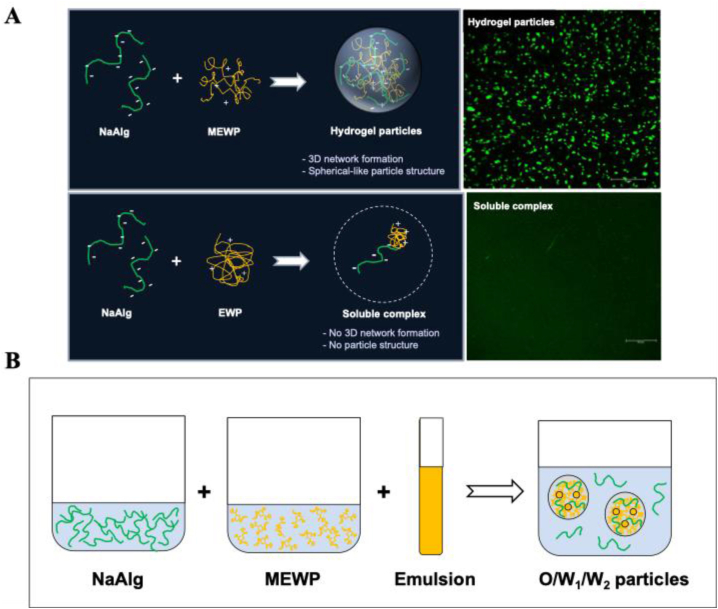
(A) Schematic illustrations and confocal images of hydrogel particles and soluble complex formed by NaAlg-MEWP and NaAlg-EWP. (B) Schematic illustration of the formation of oil droplets embedded in a MEWP-rich inner hydrogel particle phase (W_1_), dispersed in an alginate-rich continuous phase (W_2_).

Overall, our structural characterization studies suggest that the flexible and loose configuration of proteins appears to be critical in their ability to form complex coacervation with polysaccharides. Altering the compact globular conformation of EWP to create more flexible structures enhances electrostatic interactions between the modified EWP (MEWP) and NaAlg, thereby facilitating the formation of hydrogel particles (Fig. 6).

### 3.4 The protective effect of filled hydrogel particles against the lipid oxidation

**Fig. 7:**
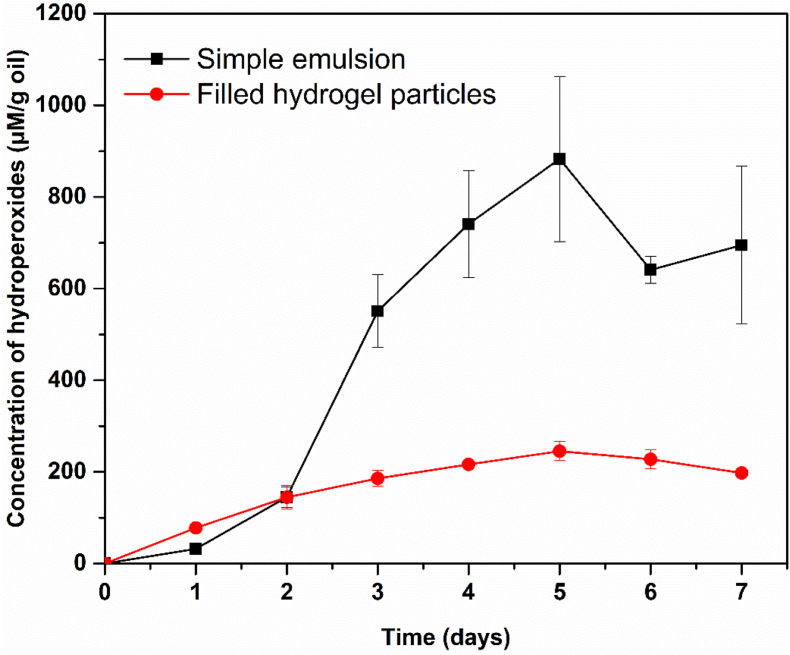
Concentration of hydroperoxides formed from fish oil during storage in the simple O/W emulsion and filled hydrogel particles.

Hydroperoxides are the primary oxidation products, and their formation and accumulation serve as significant indicators of lipid oxidation (Jaldani et al., 2024). As shown in Fig. 7, the concentration of hydroperoxides in the simple emulsion sharply increased after two days of storage, followed by a subsequent decline, which is a typical oxidation behavior associated with the decomposition of primary oxidation products into secondary oxidation compounds. In contrast, the production of hydroperoxides in the filled hydrogel particles was significantly lower during storage. Moreover, the rapid formation of hydroperoxides, typical of the chain propagation phase, was not observed in the filled hydrogel particles. During the chain propagation phase, the exponential increase in free radicals typically causes a sharp rise in hydroperoxides formation rate (St Angelo, 1996). As secondary oxidation products originate from the decomposition of primary hydroperoxides, the suppressed formation of hydroperoxides in the filled hydrogel particles inherently limits the subsequent generation of aldehydes, ketones, and other secondary oxidation products. In contrast, the accumulation and subsequent decline of hydroperoxides in the simple emulsion reflect their conversion into secondary products, which is a well-established feature of lipid autoxidation. Collectively, this suppressed propagation behavior suggests that the hydrogel matrix interferes with the oxidation cascade at an early stage, thereby attenuating the overall oxidative deterioration.

Three factors may account for these observed results: (1) The encapsulation of fish oil reduced its direct exposure to oxygen. Unlike conventional O/W emulsions, in which oil droplets are directly exposed to the aqueous phase, the O/W_1_/W_2_ architecture physically confines the oil droplets within a protein–alginate hydrogel matrix. This matrix acts as a dense diffusion barrier that limits oxygen transport and restricts the mobility of reactive species. As discussed earlier, the filled hydrogel particles possess a denser and less swollen network structure, which greatly reduces the effective diffusivity of oxygen. (2) Both EWP and MEWP exhibited strong antioxidant properties (Supplementary Fig. S1). Notably, modified MEWP showed significantly enhanced antioxidant activity, which may be attributed to the exposure of active amino acid side chains (such as tyrosine, tryptophan, and histidine) induced by pH and temperature modifications (3) The hydrogel particles may inhibit free radical propagation in the system through physical compartmentalization. Specifically, the spatial confinement of oil droplets within the hydrogel matrix restricts the transfer of lipid radicals and peroxyl radicals between neighboring droplets, thereby suppressing inter-droplet chain reactions. Moreover, as discussed earlier, the protein–alginate matrix forms a compact and non-swelling network, which further limits molecular mobility within the system and reduces the probability of radical–radical encounters.

**Fig. 8:**
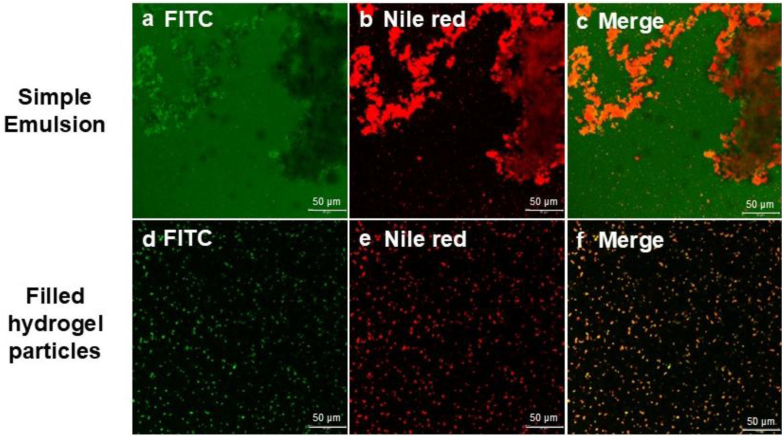
(a–c) Simple emulsion: proteins were stained with FITC (green, a), oil droplets were stained with Nile Red (red, b), and the merged image is shown in (c). (d–f) Filled hydrogel particles: proteins were stained with FITC (green, d), oil droplets were stained with Nile Red (red, e), and the merged image is shown in (f).

In addition to affecting the nutritional properties of emulsions, lipid oxidation can also alter their interfacial properties (Ghelichi et al., 2023; Waraho et al., 2011). As shown in Fig. 8, fish oil in the simple emulsion aggregated into large, irregular clusters after storage, whereas the filled hydrogel particles retained their original morphology. These results indicated that encapsulating fish oil in hydrogel particles not only improved its oxidative stability but also preserved its dispersion stability.

## 4 Conclusions

This study demonstrated that the MEWP obtained from pH 13 + 50 °C treatment could form micrometer-sized and uniform shaped hydrogel particles with NaAlg through electrostatic complexation. This could be attributed to the increased surface hydrophobicity and structural flexibility of EWP following structural unfolding, which promoted extensive electrostatic interactions between the newly exposed hydrophobic regions of MEWP and NaAlg at pH 4.5. The fish oil emulsions could be encapsulated within the MEWP-rich dispersed phase, and their incorporation into hydrogel particles effectively protected them against lipid oxidation by providing a physical barrier and an antioxidant-rich environment created by MEWP. Additionally, the dispersion stability of the fish oil emulsion in aqueous phase was also improved. These findings have important implications for expanding the application of highly hydrophilic proteins with compact globular structures in structured emulsions.

## CRediT authorship contribution statement

Fan Zhang: Data curation, Software, Investigation, Methodology, Formal analysis, Writing - original draft, Writing - review & editing. Hangxin Zhu: Software, Methodology. Fangfang Li: Methodology. Bongkosh Vardhanabhuti: Methodology, Resources. Azlin Mustapha: Methodology, Resources. Yujie Su: Writing - review. Yanjun Yang: Supervision. Zipei Zhang: Conceptualization, Resources, Supervision, Writing – review, Funding acquisition.

## Submission declaration and verification

We declare that the work described was original research that has not been publication elsewhere.

## Declaration of competing interest

The authors confirm that they have no conflicts of interest with respect to the work described in this manuscript.

## Data Availability

Data will be made available on request.
