# Supplementary material for: Structural modification of egg white proteins enhances electrostatic complexation with sodium alginate and hydrogel particle formation: mechanistic insights and functional applications
Source: Curr Res Food Sci. 2026 Feb 3;12:101339. doi: 10.1016/j.crfs.2026.101339 (PMC12906068; doi:10.1016/j.crfs.2026.101339)
Supplement: Multimedia component 1 [file mmc1.docx]

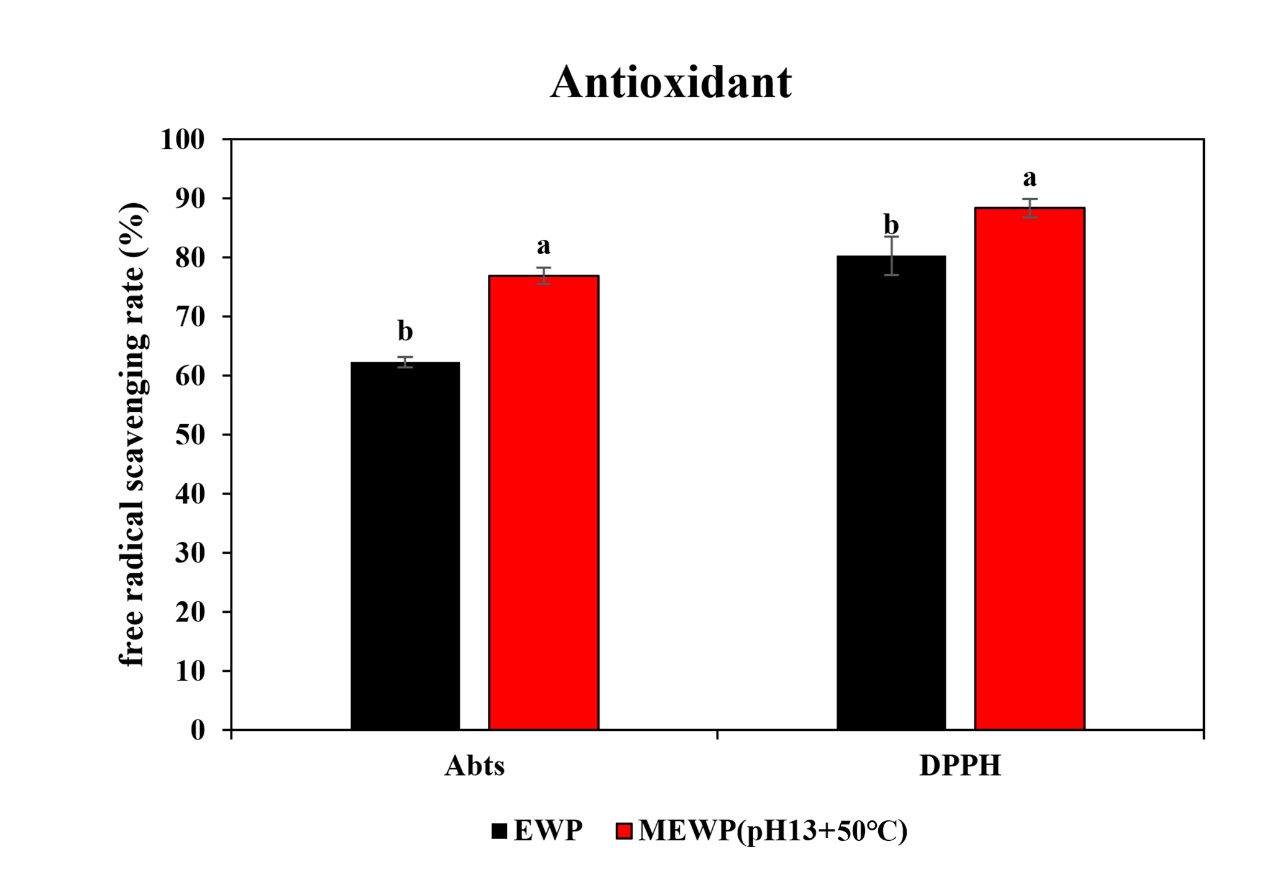


**Fig.S1** Free radical scavenging rate of EWP and MEWP (pH 13 + 50 °C) using ABTS and DPPH radical scavenging assays
